# Horizontal Tourism Coopetition Strategy for Marketing Performance – Evidence From Theme Parks

**DOI:** 10.3389/fpsyg.2022.917435

**Published:** 2022-07-19

**Authors:** Meiju Wu, Jianmin He

**Affiliations:** College of Business, Shanghai University of Finance and Economics, Shanghai, China

**Keywords:** tourism marketing, sustainable competitiveness, COVID-19 pandemic, strategic management (SM), coopetition between partners, performance

## Abstract

Unprecedentedly impacted by COVID-19, tourism enterprises are pushed to adopt new strategic management to cope with the changes in tourists' consumer perception for sustainable development, such as corporate and compete simultaneously with their competitors. Our study aims to investigate the intermediate role of coopetition, including the three dimensions of resource similarity, market commonality, and willingness to cooperate in the marketing and performance relationships. Primary data on 360 observers were collected *via* questionnaire distribution to theme park managers in China with 85.3% accuracy in response rate. Structural equation modeling (SEM) was used to verify the intermediate effect of coopetition on marketing performance in tourism enterprises. The results of SEM indicate (1) the intermediate role of tourism coopetition, including the three dimensions existing in the relationship between tourism marketing and performance, (2) more significant positive impact on non-financial performance than that on financial performance, and (3) the mechanism of implementing coopetition. This study gives supportive evidence for tourism enterprises to implement coopetition and highlights the implications for appropriately developing coopetition strategies and tactics to achieve the synergy effect for the individual enterprises and the spillover effect for the destination regarding policy-making, mindset, and partner selection.

## Introduction

Tourism industry has experienced an unprecedented crisis caused by COVID-19, which directly influences the tourists' consumer perceptions, behavioral intentions, acquisition decisions (Watson and Popescu, 2021), and even consumer satisfaction judgments (Watson and Cug, 2021). Sensitively affected by global crises and disasters (Ugur and Akbiyik, 2020), current tourism industry is faced with three main problems, lack of potential market, emerging special requirement of tourism product upgrading, and tighter marketing budgets (Nguyen et al., 2021). Tourism enterprises are pushed to adopt the new strategy as coopetition (Filimonau, 2021) in the new marketing milieu, that is to cooperate with each other even if with their partners holding clearly competing goals (Dyer et al., 2008) by sharing the resource that cannot be obtained in the market to encourage effective innovation, so that the individual tourism enterprises can create value benefited from maximizing the destination's competitiveness. Themed Entertainment Industry Leaders' Summit in 2021 proposed the theme “Innovation, Cooperation, and Win-Win” and showed that coopetition was becoming the world developing trend in the tourism industry.

Researchers have proved that enterprises are able to achieve superior performance through coopetition than collaboration or competition alone (Czakon, 2009) as cooperation can overcome resource constraints, reduce promotion costs and operational risks (van der Zee and Vanneste, 2015), and identify more market opportunities by facilitating resource sharing and codesigning marketing strategies based on the resource-based view (Barney, 1996) and transaction cost theory (Gulati, 1995). However, although some tourism enterprises want to cooperate with their competitors to achieve their short and long-term goals, they still doubt whether coopetition is advantageous in this capacity and the lack of the confidence and motivation to do so practically. This study is designed to do the empirical research on the horizontal coopetition formed between the tourism enterprises with the homogeneous product. We choose the theme park industry as the pilots due to the following reasons. First, unsurprisingly, some of the earliest adopters of tourism coopetition are theme parks. The world-known theme park cluster in Orlando, Central Florida, formed because all the following mega theme parks were benefited from neighboring to Disney World. All the theme parks in this area keep on implementing an enviably stable and healthy price coopetition strategy instead of a vicious price war (Braun and Soskin, 1999). In addition, in 1992, the number of tourists in “Splendid China” exceeded the previous year thanks to the opening of the nearby “Chinese National Village” and proposed a positive clustering effect between theme parks (Bao, 1994). Second, theme park industry is the most difficult kind of tourism attractions to operate with highly-cooperation and highly-competition, which meets the ideal empirical context for studying coopetition (Bengtsson and Kock, 2014; Granata et al., 2018). Theme park industry is experiencing so speeding changes in developing and competitive environment that it is now in need of reorganizing their competition patterns. Third, theme parks are compulsory of continuous innovation as they are the relative luxury tourism products (Song et al., 2009), providing new and diverse vacation experiences and offering the convenience of all-inclusive journey (Yusof et al., 2021). However, continuous innovation is the heavy burden for the individual theme park being affected by the disaster and faced with budget cut-downs for both marketing and employment. In addition, as the distinguished element to mold the destination image (Lin et al., 2007), certain theme parks have cobranding (Uggla, 2004) to create the ultimate cross-marketing opportunity and build real-life experiences that immerse visitors deeper into the brands or intellectual property to achieve higher synergy effects for themselves and spillover effects for the destination.

We construct the model based on the marketing mix, including product, price, place, and promotion strategy, implemented by theme parks in China and explore the relationship between tourism marketing and performance under the intermediate effect of coopetition. As American Theme Entertainment Association (2021) announced that “China has become a global leader in themed experience development, with significant, continued growth in the region,” this study gives the supportive evidence from the research on the promising market to strengthen the confidence of improving marketing performance by adopting coopetition strategy and provides new insights into answering the following questions: (1) What kind of partner is more suitable to implement coopetition strategy with? (2) which dimensions of tourism marketing are the most effective to implement coopetition? and (3) which dimensions of performance are most significantly affected by tourism coopetition?

## Literature Review

### Tourism Marketing

Deeply influenced by the external forces, tourism marketing strategies are still the media to connect tourism enterprises and tourists by allocating and coordinating resources in marketing activities (Tielung and Untu, 2021). Marketing mix is the most common and effective strategy for tourism enterprises (Kotler and Rath, 1984) as it has joint effects on costumer's satisfaction (Tielung and Untu, 2021), as well as companies' revenues and value growth (Blut et al., 2018) in the tourism industry under risk crisis settings (Alananzeh et al., 2018). McCarthy (1960) put forward that marketing mix, including product, price, place, and promotion strategies as a set of controllable variables, to satisfy the market and pursue marketing targets. The product strategy focuses more on diverse and innovative designs to meet the needs of visitors from different market segments and build up the brand (Pikkemaat and Schuckert, 2007). The price strategy is to formulate and adjust prices so as to obtain funding and maximize revenue (Othman et al., 2020), based on scenic area and level, management system, product type, and industrial and socioeconomic environment (He, 2007). The place strategy is designed to sell tourism products by focusing on the length and width, stability, and smoothness of the controlled channel, among which the direct marketing channels have been proved to attract more consumers with brand loyalty (Thomas, 2007). The promotion strategy uses a set of information through a persuasive communication process to stimulate and arouse consumers' desire to buy, including advertising, discount promotions, public relations, personal marketing, and direct marketing (Kotler and Lee, 2008).

### Tourism Coopetition

Since first raised by Brandenburger and Nalebuff (1996), coopetition has been the subject of an increasing amount of research applied to tourism industry sectors. Scholars argued that competitive and collaborative strategies were utilized simultaneously by enterprises from the perspective of strategic management, behavioral, and game theory (Chen, 2008; Crick et al., 2022; Nguyen et al., 2022). We make the literature review based on the classification of different levels of coopetition applied in the tourism industry put forward by Chim-Miki and Batista-Canino (2017b) (shown in Table 1).

**Table 1:** Different levels and dimensions of coopetition applied in the tourism industry.

**Coopetition**	**Definition**	**Application in the tourism industry**
Micro coopetition (intra-organizational level)	It refers to the function or divisions within an organization compete for enhancing their product and service efficiency Dagnino and Padula (2002).	Not found in tourism literature.
Meso coopetition (inter-organizational level in horizontal dimension)	It consists of firms involved in synergistic networks where their efforts are supported by external and institutional factors, most of which achieve competitive advantage from knowledge sharing (Chim-Miki and Batista-Canino, 2017a).	Kylanen and Mariani (2012) studied on Finnish and Italian theme parks and found that competing companies tended to collaborate for marketing during low season for survival with more egoistic attitudes, however, they were seldom corporate during peak season.
		Titmas (2012) studied the coopetitive relationship between six hotels in Cape Town, South Africa, and found that coopetition was affected by the external factors, including geographic proximity, economic climate, the role of a third party, and the internal factors, including management and ownership, clear organizational goals, communication skills among leadership, and management structure.
		Crick (2018) proved coopetition to be a strategy to increase performance by interviewing 25 firms competing in the New Zealand wine industry.
Macro coopetition (inter-network level in both horizontal and vertical dimension)	It concentrates on competitive relationships between companies or destinations supported mainly by the theories of competitiveness with comarketing, social inclusion programs, training for tourism infrastructure improvements, and joint solutions to local problems Chim-Miki and Batista-Canino, 2017b.	Kylänen and Rusko (2011) studied on coopetition between firms and the public sector in Finland and found that competitors usually competed at the destination level but collaborated at the international level.
		Wang and Krakover (2008) explored the business relationships among the stakeholders in the destination conducting collaborative marketing activities in tourism industry and verified the coexisting of cooperation, competition and coopetition among the tourism stakeholders to achieve success for both the individual and destination.
		Guo et al. (2014) made an economic game analysis of an online supply chain, including a hotel and an online travel agency (OTA) and proposed the optimal model to determine the commission fee for the hotel, and the cash back amount for the OTA.
		Lorgnier and Su (2014) studied the effects of coopetition in the context of complementary heterogeneous resources within the coopetition network to explain how to create value at the network level and confirmed that resources pooling enhanced financial performance and economies of scale.
		Kirillova et al. (2020) explored coopetition for destination brand in Greater Bay Area and found that coopetition was members into the regional brand while benefit for developing and maintaining their competitive positions within the region.
Meta coopetition (regional level in vertical dimension)	It improves the capacity and competitiveness of different groups of stakeholders to articulate their interest in the societal level to create favorable conditions for economic and social development by establishing political and economic patterns (Bouncken and Kraus, 2013).	Werner et al. (2015) found that Regional Tourism Organizations collaborated nationally while competing for visitors' spending and nights during the event of the 2011 Rugby World Cup.
		Sirisuthikul (2018) suggested that ASEAN tourism should adopt a coopetition framework and cocreate a consistent and coherent positioning of the destination, while maintaining a competitive positioning of the nation to achieve a sustainable brand of ASEAN tourism.

*Compiled by authors*.

### Tourism Marketing Coopetition

As a fundamental firm marketing strategy, cooperation in marketing activities shows the implications for business strategies (Crick, 2020). It determines the competitiveness of tourist destinations by integrated management (Chim-Miki et al., 2020), and it also enhances value creation and stimulates an innovation of tourism enterprises (Della Corte and Sciarelli, 2012) by the positive effect on the tourism area life cycle (Das and Teng, 2001), as well as the resource sharing and cost-saving mechanism (Chim-Miki and Batista-Canino, 2017a). The researches focusing on how to change one aspect of the marketing mix using coopetition strategies have emerged rapidly after the outbreak of COVID-19, including the product innovation strategy (Burström et al., 2022), transparency and coherency communications improvement in place strategy (Choi and Powers, 2021), comarketing through resources sharing (Crick and Crick, 2020), and the promotion of local culture in low season (Dewa Rucika et al., 2021).

### Tourism Coopetition and Performance

The research on the impact of coopetition performance is an essential question (Le Roy and Czakon, 2016), which is primarily on the value created by the collaboration advantages based on the relational view (Barney, 1991) and sustainable competitive advantage view (Belderbos et al., 2004; Neyens et al., 2010; Le Roy et al., 2016). Previous researches have established that the effect of coopetition on performance is the significant strategy in the tourism industry on economic, financial, and market performance (Oum et al., 2004; Lemmetyinen and Go, 2009; Della Corte and Aria, 2016; Crick et al., 2021; Le Roy et al., 2022). Coopetition strategy has proved to stimulate service innovation through the moderating role of coopetition recognition (Wang and Chen, 2021) and to improve the exploration, access, leverage, and exploitation of resources across firm (Kallmuenzer et al., 2021). The attraction of tourism coopetition lies in the commitment to achieve excellent performance through high cooperation in a highly competitive environment. The key drivers of high cooperation are in two aspects: the greater sharing of resources and capabilities with competitors through formal and informal means, i.e., resource similarity; and the degree of cooperation intention based on the trade-offs and judgments of the pros and cons of cooperative behavior (Gnyawali and Park, 2011), i.e., willingness to cooperate (Chen, 1996). High competition refers to competitive incentives with competitors in similar markets, so market commonality becomes the basis of competition (Chen, 2008).

## Methods

### Hypotheses Development

We develop the quantitative methods used by Al-Qarni et al. (2013) and Mintz and Currim (2013) to evaluate the influence of marketing mix on performance and explain the variables in Table 2.

**Table 2:** Description of variables.

**Variables**	**Item**	**Symbol**	**Details**
Dependent variable	tourism marketing	Mkt	Expressed by marketing mix, including product, price, place, and promotion strategies.
Independent variable	Tourism performance	Perf	Including the financial and non-financial performance of tourism enterprises.
Intermediate variables	Resource similarity	RS	Referring to the degree to which a firm is similar to a competitor in terms of the number and type of resources.
	Willingness to cooperate	WC	Referring to the difference between gains and losses depending on how people define a situation.
	Market commonality	MC	Referring to the extent to which there is overlap in the target markets that the competitors are competing for.

*Compiled by authors*.

The dependent variable is the marketing mix, including product, price, place, and promotion strategies McCarthy (1964). The independent variable is derived from the previous results, including the financial and non-financial performance of tourism enterprises. Financial performance includes profitability (Wincent et al., 2010), operational capabilities, and solvency (Bornhorst et al., 2010), whereas non-financial performance includes tourist satisfaction (Kozak, 2002), innovation capability, market share capacity (Niavis and Tsiotas, 2019), anti-risk ability (Hongqing and Wei, 2019), operating cost efficiency (Chai et al., 2022), and ability to achieve sustainable development (Ritchie et al., 2001). The intermediate variables are tourism coopetition that comprised of three dimensions in these current studies, including resource similarity (RS), willingness to cooperate (WC), and market commonality (MC), which are derived from the empirical analysis of the tourism industry of Chen (1996, 2008).

A comprehensive and effective marketing strategy is composed of product, price, place, and promotion strategies, which has an indispensable impact on the performance of tourism enterprises. For example, product upgrading and new product development strategies are fundamental for marketing penetration (Benur and Bramwell, 2015). Based on the product strategy, the effective pricing strategy and flexible price adjustment strategies are required according to product characteristics, market demand, and competition, so as to maximize the income of the enterprise (Heo and Lee, 2009). In this process, promotion strategies help enterprises awake travel motivation, stimulate desire, and promote purchase through diversified channels, so as to finally achieve better performance (Amin and Priansah, 2019).

**H1:** If a tourism enterprise can set a comprehensive and powerful marketing strategy involving product, price, place, and promotions, it can achieve a significant performance improvement.

From a resource-based view, competitors have similar resources and capabilities because they operate in overlapping markets centering on comparable products (Ko et al., 2020). From the existing research, resource similarity is derived from several factors: (1) similarity in the quantity and types of internal resources between an enterprise and its competitors (Jayachandran et al., 1999); (2) similarity in the forces to achieve common goals from both sides (Cole and Bruno Teboul, 2004); and (3) similarity in the requirement asked for the competing parties (Ndubisi, 2011). Previous empirical research have proved the effect of RS on performance from different dimensions, such as RS can foster coopetition as unique resources to be advantageous for cooperation and competition (Bengtsson and Kock, 2000) and affect the coopetition stability and performance (Raue and Wallenburg, 2013). It is proved to reduce the opportunism behavior and the incompatibility of partners Brekalo and Albers (2016), which may lead to the coopetition instability because of the lack of trust and higher transaction costs (Prashant and Harbir, 2009), create value by opening up new markets through integration, and become more profitable to optimize the performance (Estrada and Dong, 2020).

**H2:** RS has an intermediate effect on the relationship between tourism marketing and performance, so that the higher RS is, the stronger becomes the relationship.

Willingness to cooperate is based on the trade-offs and judgments of the pros and cons of cooperative behavior (Gnyawali and Park, 2011). From the existing research, willingness to cooperate is derived from several factors: (1) corporate orientation, including the corporate level for the whole destination (Bounckenm and Fredrich, 2012) and the closeness of enterprises (Kotzab and Teller, 2003); (2) corporate experience, including the experience between the candidate enterprises (Gnyawali and Park, 2009) and among the other enterprises (Mariani, 2016); (3) perceived benefit, including mutual beneficial action (Della Corte and Aria, 2016), information sharing (Fernandez and Chiambaretto, 2016), value creation (Bagdoniene and Hopeniene, 2015), and comarketing (Pranjal and Sarkar, 2020); (4) reputation, including the actual and evaluated reputation of the other party (Czernek et al., 2017); (5) trust in the partnership (Kraus et al., 2018) and the coopetition project (Raza-Ullah and Kostis, 2020); and (6) strategic fit (Czakon and Czernek, 2016), especially collaborate to pursue a common goal (Bouncken et al., 2015).

**H3:** WC has an intermediate effect on the relationship between tourism marketing and performance, such that the higher is WC is, the stronger becomes the relationship.

Market commonality arises between enterprises that provide similar products and services in the same market (Porter, 1986), which refers to the degree of competition in the common market (Chen, 1996; Derfus et al., 2008). Understood as the same degree of market quantity and importance between enterprises and competitors, market commonality can influence the degree of competition and upgrade competitive advantage (Chen, 1996).

Scholars usually testify and verify the relationship between RS, MC, and performance. Derfus et al. (2008) put forward that enterprises usually spent more time monitoring rivals' strategic behaviors and were inclined to tolerate each other and acquiesce employing no strategic behavior to avoid ultimate loss when market commonality reaches a certain level. Kotler and Keller (2016) pointed out that enterprises can jointly study the demand preferences of tourists, create new products to meet their needs, and adopt joint marketing activities when facing a similar demand curve. Schwartz and Webb (2021) adopted interquartile to measure the variability of RS, MC, and performance and the difference between the quartile and found that the higher MC is, the stronger becomes the relationship between marketing and performance. Czakon and Czernek-Marszalek (2021) adopted qualitative research to explore how managers' cognitive maps are linked to competitor identification mainly referring to MC.

**H4:** Market commonality has an intermediate effect on the relationship between tourism marketing and performance, so that the higher MC is, the stronger becomes the relationship.

**Figure 1:**
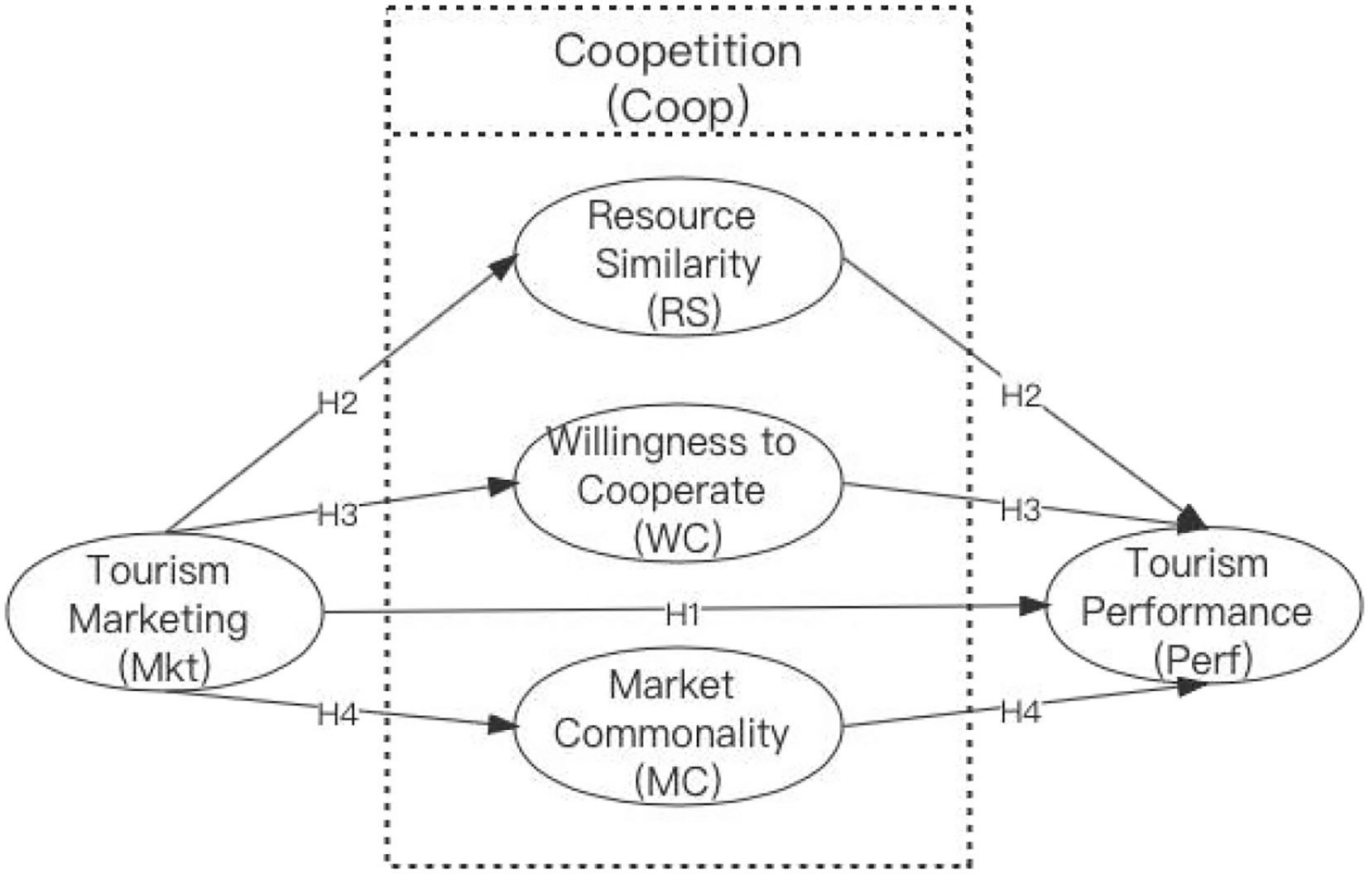
Proposed theoretical framework.

According to the hypothesis, the proposed theoretical framework is illustrated in Figure 1.

### Data and Sampling

Primary data are widely used to observe tourism enterprises' coopetition behaviors and relationships. Most of the factors put forward by Leask and Fyall's (2008), which have a significant effect on tourism coopetition, are linked to managers. As such, we designed a questionnaire to be answered by the current or former managers and related staff of theme parks in China, to ensure the representative and coverage of the sample and the rationality, effectiveness, and reliability of this study. The questionnaire was designed to determine the status of marketing, coopetition, and performance of the theme parks through anonymous scoring.

We first conducted three semi-structured interviews with theme park managers having comprehensive marketing and managerial knowledge. Then, we set up the indicators and questions based on the literature review, accompanied by professional interviews. We invited three experts to complete the preliminary questionnaire based on their own experiences and asked for suggestions to better adapt the analysis to tourism marketing, coopetition, and performance. We used an electronic survey (through Questionnaire Star) as the substantive research method. All the answers were collected on a five-point Likert-type scale rating the extent to which a respondent either agreed or disagreed with the statements. Once the survey was designed, it was pretested with a sample of theme park managers (*n* = 10) who could comment on the constructs being measured. The pretest stage did not reveal any concerns, and a pilot study was undertaken (*n* = 112). No changes were made to the survey based on the feedback from the pilot study. Next, a core study was conducted (*n* = 238) from the population of 300 instructors. We removed the invalid questionnaires from analysis, that is, those that provided the same scores to all the questions. As the measures in the pilot study were identical to those in the core study, the data sets were merged to yield the final sample (Crick, 2021), and we have totally 360 observations accounting for an acceptable response rate (85.3%) to test the proposed model.

### Measures

We followed a procedure similar to the survey designation and conduction made by Rai (2016). For new scales, the measurement variables were generated through a literature review, followed by several tests, such as professional review, pretest, and liability assessments. The adopted method is structural equation modeling, a well-known technique for studying relationships among multivariate data.

Reliability was assessed using Cronbach's alpha coefficients (α) to check the consistency of variables for each measurement item, which requires that all values should be above 0.70.

## Results

### Reliability, Validity, and Common Method Bias

Table 3 lists the reliability and convergent validity values. First, we analyzed the individual indicator reliability, internal consistency, and convergent validity to assess the soundness of the measurement model. The standard factor loading of the items onto their corresponding constructs was all >0.70, indicating a high degree of individual indicator reliability. All composite reliability values were higher than the minimum threshold of 0.70, which further established internal consistency reliability. To assess convergent validity, we examined the average variance extracted (AVE), which was above the recommended limit of 0.50 for all constructs.

**Table 3:** Constructs, indicators, and reliability.

**Construct**	**Indicators**	**SFL**	**α**	**CR**	**AVE**
Performance (Perf)	In this relationship, the following indicators have been improved significantly:		0.821	0.821	0.605
	Financial performance (e.g., profitability, operational capabilities, solvency)	0.799		
	Non-financial performance (e.g., tourist satisfaction, innovation capability, market share of capacity, anti-risk ability, operating costs efficiency, ability to achieve sustainable development)	0.743			
Marketing (Mkt)	In this relationship, the overall strategy is developed based on:		0.82	0.824	0.611
	Product (e.g., life-cycle theory based, a completely new product development, and regular product upgrading strategy)	0.7			
	Price (e.g., low demand price elasticity, comprehensive pricing system, stable law of price adjustment, slow down frequency of price changes)	0.789			
	Place (e.g., on-site, Internet, multilevel and multi-tier, intermediaries-removing distribution channels)	0.849			
	Promotion (e.g., disseminate information, stimulate demand, buildup brand image)	0.813			
Willingness to cooperate (WC)	In this relationship, both parties:		0.914	0.915	0.643
	Hold a win-win attitude to break the concept of gain or loss	0.829			
	Do not care about the pay-as-you-get-back-to-the-other relationship	0.805			
	Are willing to share knowledge and invest resources with partners	0.743			
	Fully regard the partner as trustful	0.832			
	Can communicate with partners smoothly	0.778			
	Highly match the partner's strategy goal	0.819			
Resource Similarity (RS)	In this relationship, both parties:		0.825	0.827	0.615
	Have a high degree of similarity in quantity and type of competitor	0.846			
	Are willing and able to provide the resources meeting each other's needs	0.778			
	Can make use of complementary resources from the partner	0.723			
Market Commonality (MC)	In this relationship, both parties:		0.828	0.829	0.618
	Are in the same industry which is highly competitive	0.779			
	Provide homogeneous products or services	0.754			
	Face the same source market	0.824			

^*^p < 0.001.

*SFL, standard factor loading*.

*α, Cronbach's alpha*.

*CR, composite reliability*.

*AVE, average variance extracted*.

Second, we used SPSS 23.0 for exploratory factor analysis as KMO and Bartlett's spherical test in Table 4. KMO was >0.7 and Bartlett's spherical test value was significant (*p* < 0.001). All the results indicated that the questionnaire data met the prerequisite requirements for factor analysis.

**Table 4:** KMO and Bartley's sphere test.

KMO measure of sampling adequacy		0.828
Bartlett 's test	Approx. Chi-square	863.435
	df	10
	*p*	0.000

Third, we used SPSS 23.0 to perform the confirmatory factor model fit test, and the results shown in Table 5 indicated that the model fitted into the general research criteria, so it could be considered to have a good fitness.

**Table 5:** Model fit test.

**Indicators**	**χ^2^**	**Df**	**χ^2^/df**	**GFI**	**RMSEA**	**RMR**	**CFI**	**NNFI**	**NFI**
Recommended value	–	–	<3	>0.9	<0.10	<0.05	>0.9	>0.9	>0.9
Actual value	9.174	3	3.058	0.936	0.332	0.112	0.898	0.916	0.918

Fourth, we used a more rigorous AVE method to evaluate the effectiveness of discriminant validity. Discriminant validity exists when the AVE root number of each factor is greater than the correlation coefficient of each pair of variables. As shown in Table 6, the AVE root number of each factor was greater than the standardized correlation coefficient outside the diagonal, and this study had discriminant validity with the correlation coefficient as a slanted down triangle.

**Table 6:** Discriminant validity test.

	**Mkt**	**WC**	**RS**	**MC**	**Perf**
Mkt	0.782				
WC	0.374^**^	0.802			
RS	0.439^**^	0.420^**^	0.784		
MC	0.410^**^	0.520^**^	0.431^**^	0.786	
Perf	0.585^**^	0.609^**^	0.570^**^	0.575^**^	0.778

*p < 0.05;

** *p < 0.01*.

### Hypothesis Testing

Table 7 provides a detailed summary of the structural model results. We conducted three types of regressions. The first type included the independent variable marketing and the dependent variable performance. The second type included the independent and the intermediate variables. The third type included all three variables.

**Table 7:** Results of regression models.

	**Perf**					**WC**					**RS**					**MC**					**Perf**				
	**B**	**SE**	**t**	**p**	**β**	**B**	**SE**	**t**	**p**	**β**	**B**	**SE**	**t**	**p**	**β**	**B**	**SE**	**t**	**p**	**β**	**B**	**SE**	**t**	**p**	**β**
	0.197	0.161	1.223	0.222	–	1.306^**^	0.223	5.851	0	–	1.128^**^	0.235	4.791	0	–	0.911^**^	0.233	3.907	0	–	−0.459^**^	0.14	−3.283	0.001	–
Mkt	0.958^**^	0.043	22.474	0	0.765	0.632^**^	0.059	10.705	0	0.492	0.703^**^	0.062	11.289	0	0.512	0.732^**^	0.062	11.867	0	0.531	0.567^**^	0.045	12.533	0	0.453
WC																					0.222^**^	0.034	6.62	0	0.228
RS																					0.213^**^	0.031	6.977	0	0.234
MC																					0.137^**^	0.032	4.283	0	0.151
*R* ^2^	0.585					0.242					0.263					0.282					0.728				
Adjusted *R*^2^	0.584					0.24					0.26					0.28					0.725				
*F*-value	*F*_(1, 358)_ = 505.062, *p =* 0.000					*F*_(1, 358)_ = 114.590, *p =* 0.000					*F*_(1, 358)_ = 127.430, *p =* 0.000					*F*_(1, 358)_ = 140.826, *p =* 0.000					*F*_(4, 355)_ = 237.500, *p =* 0.000				

*p < 0.05;

** *p < 0.01*.

From Table 5, we had five models for the intermediate effect:

Perf = 0.197 + 0.958 ^*^ Mkt,

WC = 1.306 + 0.632 ^*^ Mkt,

RS = 1.128 + 0.703 ^*^ Mkt,

MC = 0.911 + 0.732 ^*^ Mkt,

Perf = −0.459 + 0.567 ^*^ Mkt + 0.222 ^*^ WC + 0.213 ^*^ RS + 0.137 ^*^ MC.

We obtained the results of the intermediate effect from Table 8. The results for a, b, and c′ were all significant, showing that the intermediate effect of the willingness to cooperate, resource similarity, and market commonality was partial.

**Table 8:** Results for the intermediate effect.

**Item**	**Result**	***c***	***a***	***b***	*a* *b	***c* ^**′**^**	*a* *b/*c* (%)
Mkt => WC => perf	Partial	0.958	0.632 ^**^	0.222 ^**^	0.141	0.567	14.664
mkt => RS => perf	Partial	0.958	0.703 ^**^	0.213 ^**^	0.15	0.567	15.641
mkt => MC => perf	Partial	0.958	0.732 ^**^	0.137 ^**^	0.101	0.567	10.502

*p < 0.05;

** *p < 0.01*.

c is the total effect representing the regression coefficient of X to Y (when there is a mediation variable M included in the model), a represents the regression coefficient from X to M, b represents the regression coefficient from M to Y, a ^*^ b is the intermediate effect, and c' is the direct effect representing the regression coefficient of X to Y (when there is a mediation variable M included in the model).

### Discussion

#### The Positive Links Between Tourism Marketing and Performance

These findings may help us to understand that the well-designed product, price, place, and promotion strategies play a continuously significant role in financial and non-financial performance in the actual market operation process. The result is complied with the prior studies that noted the importance of tourism marketing to optimize performance (Della Corte and Sciarelli, 2012).

#### The Positive Intermediate Effect of Tourism Coopetition on the Relationship Between Marketing and Performance

Resource similarity directly affects the likelihood and extent to which tourism enterprises can obtain resources from their competitors who are eager to maintain their competence to make profit (Lee and Fong, 2016). Entrepreneurs can learn how to understand the competitive environments better through coopetition and gain the advantages from acquiring new resources, capabilities, and opportunities, which could not been obtained by individual effort (Bouncken and Kraus, 2013; Hannah and Eisenhardt, 2018; Kraus et al., 2018). The nature of coopetition is also influenced by the degree of the willingness to cooperate, including common goal, mutual goodwill, trust, former experience, and perceived benefit. An interesting finding is the intermediate role of market commonality which is also significantly positive. The analyzed samples showed their intention to cooperate with direct rivals offering homogeneous products or services and facing the same target market. Because of the huge investment and high risk, tourism enterprises have burden on continuous innovation and difficulties in attracting international tourists using push-and-pull promotional strategies alone. Adapting to the increasingly competitive environment, tourism enterprises that have similar target markets combine their competitive resources that cannot be obtained from the open market to develop joint marketing and innovation to reduce the cost and create value through mergers and acquisitions, restructuring, strategic alliances, and cooperation agreements. If there is no common market, tourism enterprises cannot set uniform strategic goals and support long-term stable coopetition to improve performance. Thus, coopetition has led to more effective and efficient destination marketing strategies, more complex and appealing products, more flexible and mutual benefit pricing systems, and more successful attractions for existing and potential visitors.

#### The Mechanism of Implementing Tourism Coopetition

These findings raise intriguing questions regarding the mechanism of implementing tourism coopetition, including the drivers, the participants, and the impact. As the pandemic directly influences the visitors' behavior reflected as stressed, anxious, depressed, hurt, and worried (Nguyen et al., 2021), tourism enterprises should grasp which are the most effective strategic marketing action. From the study, the results comply with the findings of Bengtsson and Kock (2000) that (1) enterprises compete in activities close to the customer and cooperate in activities far from the customers and (2) enterprises compete at the destination level but collaborate at the international level. When developing marketing mix for tourism enterprises, the traditional on-site distribution channels, the all-encompassing pricing system concerning the price elasticity, the product upgrading and the promotion highlighting competitive advantage are the most effective drivers, leading to a more significant impact on the performance under the intermediate role of coopetition. For instance, the tourism enterprises that are highly depending on the third party platforms are moving to upgrade their own booking systems and websites to serve the visitors and collect the sales data directly, so that they can improve the profit.

Since each partner benefits if all rivals reciprocate (Kraus et al., 2018), the fundamental step for implementing tourism coopetition is to look for the ideal partner. From the empirical study, we find that the prestige participant is the one who holds the win-win attitude and strong trust, has the high degree similarity in the resource scale and type, and faces the relatively same market. What is more, the strategic fit, willingness to share knowledge and invest resources, and the ability to communicate smoothly are positive to implement coopetition as it lessens the opportunism behavior. It is the same as the practical implementation in the hotel industry and airline industry that coopetition behavior should be acted between the leading companies.

What is more, when implementing coopetition, the impact on non-financial performance is more significant than on financial performance. The biggest impact is significantly enhanced capacity to achieve sustainable development for both parties. Coopetition also positively affects the market occupancy capacity and the ability to make profitability. However, it has minor effect on cutting down the operational risks and operating costs. The results verify that coopetition outcome is moving from pure cost-saving to innovative solution which is more important to the tourism enterprises for sustainable development (Zacharia et al., 2019).

## Conclusion and Implications

### Conclusion

Under the pressure of pandemic, tourists are hesitated to engage in travel activities due to the perceived risks, the budget, and the restriction. Severe changes have occurred and will have continual impacts on the competitive environment for entities in the tourism and leisure industries. Under resource-based view and transaction cost theory, this study explores certain firm level- and strategic-level competitive forces that affect the relationship between marketing and performance with the intermediate effect of tourism coopetition. Through empirical research, it opens the black box of how coopetition can lead to improved marketing performance, especially under the conditions of resource similarity, market commonality, and high willingness to cooperate. The results emphasize that a competitive business environment can affect the performance outcomes of coopetition strategies in different ways and partially address the prerequisites of partners. This not only highlights the importance of the willingness to cooperate and resource similarity between rivals, which are the keys to maintain a good partnership, but also raises the issue that there must be some common markets between competing parties. Coopetition benefits otherwise unattainable are as follows: (1) developing new products and upgrading existing products through cooperative R&D; (2) improving the stable and effective pricing system to avoiding price war at the expense of revenue; (3) weakening the role of the intermediary channels and broadening channels by taking cooperative place strategy action to enhance government bargaining power and market penetration; and (4) creating and winning the market through joint advertising, coorganizing public welfare activities, and other promotional activities.

Tourism enterprises have heavy burden on the requirement of highly innovated marketing brought about by the sharp increase in the homogeneity of products from the same destination and realize that they can discover potential capacity and create a larger market based on cooperative strategic behaviors toward their rivals. Specifically, in the era of post-pandemic, for under-resourced enterprises potentially facing survival-related challenges, employing coopetition can help them to sense and seize the opportunities to improve the performance. Tourism enterprises are encouraged to scale up cooperation agreements with their rivals on innovation and comarketing, so that they can employ same expertise, capabilities, and reputation (Dussauge et al., 2000), to keep the sustainable advantages by obtaining new resources and capabilities (Felzensztein et al., 2018), and to leverage resources using the ability to access, absorb, and innovate resources to increase profit (Morris et al., 2002).

### Implications

How to rationally choose and implement coopetitive strategic behavior to obtain better performance has become an urgent problem to be solved. The managerial implications could benefit the sustainable development of tourism industry.

The industry-wide cooperative mindsets are required by tourism enterprises (Ritala and Tidström, 2014) to promote cooperative behavior by accepting the assumption that the coopetition strategy is positively related to performance. Managers must thus work together to provide complex products (services) to improve visitor satisfaction and always guard against the competitive behavior of rivals, as well to avoid opportunistic behaviors.

Giant enterprises are the preferred partners to avoid the conflict caused by the incompatible. Coopetition is not perfect and does not necessarily produce win-win results because of the opportunism behavior, especially in the case that knowledge is mostly one-way flow between the two sides in the unequal competitive position. Implement coopetition between giant tourism enterprises can (1) meet the joint innovation needs of tourism enterprises and play the synergy effect by sharing effective and compatible resources, lowering the innovative costs and risks, and improving R&D efficiency; (2) avoid excessive competition among leading enterprises in tourism market, protect the profit margins of both sides, and maintain the overall effectiveness and efficiency of tourism development in the region; and (3) improve the attractiveness of tourist destinations and play the spillover effect as the coopetition behavior helps the formation of clusters to enhance the brand effect of tourist destinations, thus expand the attraction to the international market, and create value for their own enterprises based on the expanding the existing tourism market.

It is worth noting that the supporting and supervising government policies are the triggers for tourism enterprises to adopt coopetition by being encouraged to strengthen trust-building mechanism, occupy potential markets, and create value in the optimized competitive environment. Indeed, policies can be made to create a more competitive destination by constructing regional and industry advantages, breaking the barriers of market scale expansion, seizing potential market development opportunities, and getting stronger investment support with the help of the tourism coopetitive strategic behavior.

### Limitations and Scope for Future Research

This study is pioneering in emphasizing tourism coopetition, but not as comprehensive as possible. As tourism coopetition is composed of horizontal and vertical strategic behaviors, tourism enterprises inevitably form various networks with all types of tourism stakeholders and set up different levels of competition and cooperative relationships to increase visitors' satisfaction. Therefore, research on horizontal coopetition is insufficient. Follow-up studies should thus focus on both horizontal and vertical coopetition to build a more systematic model to fully explain the implementation of coopetition.

Tourism coopetition is always paradoxical. Apart from excellent performance produced by the synergy and spillover effect of coopetition, it may also cause the irrational appropriation of the value created. Hence, it is vital to study how to implement coopetition in a balanced way. Future research will thus focus on the basis and mechanism of implementing coopetition by tourism enterprises.

## Data Availability Statement

The raw data supporting the conclusions of this article will be made available by the authors, without undue reservation.

## Author Contributions

MW and JH contributed to conception and design of the study and wrote sections of the manuscript. MW organized the database and performed the statistical analysis and wrote the first draft of the manuscript. All authors contributed to manuscript revision, read, and approved the submitted version.

## Conflict of Interest

The authors declare that the research was conducted in the absence of any commercial or financial relationships that could be construed as a potential conflict of interest.

## Publisher's Note

All claims expressed in this article are solely those of the authors and do not necessarily represent those of their affiliated organizations, or those of the publisher, the editors and the reviewers. Any product that may be evaluated in this article, or claim that may be made by its manufacturer, is not guaranteed or endorsed by the publisher.
